# Controversial Issue Session: Brace treatment in infantile scoliosis: brace treatment problems

**DOI:** 10.1186/1748-7161-7-S1-O75

**Published:** 2012-01-27

**Authors:** FJ Sánchez Pérez-Grueso

**Affiliations:** 1 Hospital de la Paz, Madrid

## 

Orthotic treatment of infantile scoliosis represents a challenge:

1. Most of them are progressive

2. Different etiologies

3. Associated diseases

4. Smaller size anatomy

5. Immature rib cage

Types:

1. Braces

• Do not provide immediate correction

• Treatment aimed to “hold” the spine and prevent further progression

• Risk of chest deformity in case of braces constricting the thorax

• Literature does not support the efficacy of braces in the treatment of infantile scoliosis

• Poor compliance (Parents)

2. Casts/Braces under traction

• Provide correction. Sometimes scoliosis can be reversed by early treatment with serial corrective plaster jackets in children with mild curves and at an early age (under age two)

• Immature rib cage often deforms before significant correction is transmitted to the spine

• May provoke skin problems

• They may reduce the deformity but not reverse it

• Main goal: delay surgery as much as possible

• Braces under traction diminish cast complications with the same efficacy in the long term

